# Fate of *Salmonella* spp. in the Fresh Soft Raw Milk Cheese during Storage at Different Temperatures

**DOI:** 10.3390/microorganisms9050938

**Published:** 2021-04-27

**Authors:** Adriana Lobacz, Justyna Zulewska

**Affiliations:** Department of Dairy Science and Quality Management, Faculty of Food Sciences, University of Warmia and Mazury, Oczapowskiego 7 Street, 10-719 Olsztyn, Poland; jzulewska@uwm.edu.pl

**Keywords:** *Salmonella* spp., unripened raw milk cheese, food safety, mathematical modeling

## Abstract

The aim of this study was to determine the survival kinetics of *Salmonella* spp. in unripened, fresh raw milk cheese during storage at 5, 15 and 25 °C. Microbiological (coliforms and *E. coli*, *S. thermophilus*, *Lactococcus* sp., total microbial count and *Enterobacteriaceae*) and physicochemical (pH and a_w_) characteristics were also determined. Two primary models were used to estimate the kinetic parameters of *Salmonella* spp., namely Weibull and Baranyi and Roberts (no lag) models. Additionally, goodness-of-fit of the primary models was assessed by calculating the R-Square and mean square error. *Salmonella* spp. growth in the unripened raw milk cheese was inhibited during storage, but nevertheless bacteria survived at 5 °C for 33 days (2.5 log cfu/g) and 15 °C for 18 days (1.8 log cfu/g). A decrease in the number of *Salmonella* spp. populations from an initial concentration 6.6 log cfu/g to below a detection limit was observed at 25 °C after 7 days of storage of contaminated cheese samples. It was concluded that the storage temperature significantly influenced the inactivation rate of *Salmonella* spp. in fresh raw milk cheese and proceeded faster at 25 °C compared to remaining storage temperatures.

## 1. Introduction

Tvarog is a fresh cheese very popular in Central and Eastern Europe. Tvarog represents a wider group of dairy products, mainly unripened cheeses produced by means of acid coagulation. The cheese is produced around the world, usually by denaturation and coagulation of milk proteins according to traditional recipes. Unripened soft cheeses together with the ripening cheeses are one of the most varied and attractive directions of milk processing. Tvarogs are commonly defined as “white cheese”, and it is difficult to find their counterpart in the world, although in terms of characteristics, similar to them are the American “pressed farmers cheese” and German “quark.” Polish tvarogs are characterized by a full, delicate, creamy taste, and a creamy, dense consistency with smooth grains [1,2,3].

The Polish dairy industry allocates annually approximately 20% of the total amount of milk for tvarog production. For many years, the consumption of tvarog cheese in Poland has been above 6 kg per capita and is significantly higher than the consumption of ripened and processed cheeses. The high attractiveness of this group of dairy products results from a long tradition of consumption, established eating habits and good availability, as well as variety of assortment and relatively low prices. The consumers appreciate the product for its high nutritional value. Tvarog cheeses are rich in nutritious proteins and minerals. Tvarog is also a rich source of easily digestible milk, fat and vitamins (mainly vitamin B). Depending on the method of milk coagulation, tvarog cheeses can be produced as acid or acid–rennet types of cheeses; however, traditional pressed tvarog is manufactured using acid coagulation. According to the definition, acid tvarog cheese is a partially dehydrated milk curd, skimmed or with standardized fat content, acidified by lactic acid bacteria or by direct addition of acid, i.e., lactic or citric. Industrial production of tvarog, including acid cheeses, is based on LAB fermentation. The proper selection of starter cultures containing LAB has a significant effect on the production process, but also influences the desired quality characteristics and the attractiveness of the final products. Unripened tvarog cheeses can be characterized by a clean, mild, slightly acidic taste and aroma, compact texture and uniform color from white to slightly creamy [4,5].

Fresh cheese such as tvarog has a quite limited shelf-life, mainly as a result of the high water content, which makes it a good environment for bacteria growth. Tvarog produced in the past had a very short shelf-life, from a few days up to one week, which was caused by rather low milk quality and manual production. Nowadays, tvarog is commercially produced using fully automated equipment, which in combination with the high hygiene of raw material and production, as well as innovative packaging systems, allows it to maintain quality characteristics for a period of 21 days. However, chilled storage conditions must be maintained through all stages of the distribution system as well as during end-user use. Interruption of cold chain storage may lead to overacidification (low acceptance by the consumer) and whey leakage.

Raw milk cheeses are one of the oldest known types of food manufactured. In comparison to chesses made of pasteurized milk, the raw milk cheeses are characterized by intense sensory qualities as a result of native bacteria activity (*Enterococcus* spp., *Lactococcus* spp., *Leuconostoc* spp. and *Lactobacillus* spp.) [6,7,8]. The milk pasteurization process contributes to the improvement of microbiological safety; however, on the other side, it has a negative effect, i.e., inactivation of protease, lipase and naturally present bacteria, which are the decisive factors shaping the unique taste of raw milk cheeses [9]. Raw milk and raw milk cheese consumption is steadily growing worldwide with the increasing demand for minimally processed, sustainable, healthy and local foods, but on the other side it also contributes to a situation where emerging and re-emerging pathogens once again represent a major food safety challenge [10]. Soft cheeses made from unpasteurized milk have been the most common vehicle of foodborne pathogens. Tvarogs, because of their high moisture content and chemical composition which constitutes an excellent medium for micro-organisms, have a limited shelf-life and require controlled refrigerated conditions during storage, distribution and sale. [11]. In European countries, cheese production and sale are subject to Regulation (EC) No. 2073/2005, and microbiological criteria vary according to the microorganism of concern [12]. To support the compliance with food safety criteria and process hygiene criteria, it is crucial to study and understand the influence of extrinsic and intrinsic factors that affect microbial behavior in ready-to-eat (RTE) foods [13]. Despite a significant number of reports on the microbiological safety of cheeses made from unpasteurized milk, the topic remains controversial. Pasteurization is mainly applied in order to ensure the microbiological safety of dairy products, but also inactivates the native enzymes present in raw milk (protease and lipase), as well as the naturally present microorganisms (*Enterococcus* spp., *Lactococcus* spp., *Leuconostoc* spp. and *Lactobacillus* spp.) that create the specific taste and aroma of cheeses made from raw milk [7,9,14,15]. The raw milk cheeses contain higher amounts of volatile compounds (carboxylic acids, alcohols and esters) as a result of native microflora activity [15,16].

Cheeses made of unpasteurized milk are considered to be microbiologically unsafe due to the possible presence of pathogenic bacteria, mainly *Listeria monocytogenes*, *Salmonella* spp., *Staphylococcus aureus* and *Escherichia coli* O157: H7, and they are the main target foodborne pathogens evaluated in fate studies performed with cheeses [7,8,13,17]. It should be emphasized that most of the research conducted so far has focused on the behavior of *Listeria monocytogenes* in pasteurized/unpasteurized cheeses, and there is a need to study the behavior of other pathogens responsible for food outbreaks in cheeses made from raw milk.

*Salmonella* is one of the four key global causes of diarrheal diseases and it causes annually 93.8 million cases of foodborne illness and 155,000 deaths [10]. Many salmonellosis outbreaks linked to the consumption of cheeses have been reported worldwide in recent years, including cheese made from raw milk, which evidences that *Salmonella* is also a pathogen of concern in these food matrices. According to European Regulation No. 2073/2005, the current criterion for *Salmonella* spp. in cheeses is “absence (non-detection) of the pathogen in 25 g of product” (*n* = 5, c = 0) for products placed on the market during their shelf-life [12]. Moreover, raw milk cheeses have been identified as a vehicle of 16 major *Salmonella* foodborne outbreaks in France in 2008 and 2018, representing a third of *Salmonella* outbreaks with an identified source [18,19]. *Salmonella* is an ubiquitous bacteria and can enter the milk and dairy environment from many sources. Nevertheless, adequate milk pasteurization and hurdles (applied in ripened hard cheeses) inactivate this pathogen, and salmonellosis outbreaks related to dairy products have mainly involved raw milk and fresh raw milk cheeses [20,21]. *Salmonella enteritidis* is one of the five most reported serovars from human salmonellosis cases acquired in the EU. In total, 926 salmonellosis food-borne outbreaks were reported by 23 EU MS in 2019, causing 9169 illnesses, 1915 hospitalizations (50.5% of all outbreak-related hospitalizations) and seven deaths. *Salmonella* caused 17.9% of all food-borne outbreaks during 2019. The vast majority (72.4%) of the salmonellosis food-borne outbreaks were caused by *S*. *enteritidis* [22].

Predictive microbiology deals with the mathematical modeling of microorganisms’ behavior in varied environments and is being applied in: challenge test, evaluation of microbiological shelf-life, prediction of the microbiological hazards connected with foods and microbiological risk assessment. Additionally, predictive modeling can be applied to fit the data of death kinetics of pathogenic microorganisms following thermal or non-conventional treatments, as well as to model survival during storage. Predictive models can be classified as: (i) primary models, which represent population density (or number) in time; (ii) secondary models, that describe the effects of environmental factors on the parameters of primary models (e.g., inactivation rate) and (iii) tertiary models, which are computer applications that predict the microbial behavior as a function of defined. Nowadays, predictive microbiology is being applied as a tool to improve food safety and quality and there are many models and statistical tools dealing with pathogens, toxins, spoilage microorganisms, thermal and non-thermal processes, etc. [23].

The aim of this study was to investigate the behavior of *Salmonella* spp. (growth, survival and inactivation) in the fresh soft raw milk cheese throughout storage at 5, 15 and 25 °C. The observed data were subjected to primary modeling (Weibull model, Baranyi and Robert’s without lag model) in order to calculate the kinetic parameters. Complex microbiological and physicochemical analyses of the studied cheese were performed during storage.

## 2. Materials and Methods

### 2.1. Fresh Unripened Raw Milk Cheese Characterization

Unripened soft cheese wheels (ca. 2 kg each) made from raw milk used for the study were purchased directly after production from the manufacturer. Cheese was produced from a non-standardized (in terms of fat content) whole milk with addition of the following ingredients: starter cultures, rennet and salt. Cheese was analyzed for *Salmonella* spp. absence according to ISO 6579:2002 (ISO, 2002). Cheese under aseptic conditions was portioned (25 g samples) and placed in sterile polyethylene VWR blender bags with non-woven polyester filter (VWR International Sp. z o.o., Gdansk, Poland).

### 2.2. Salmonella spp. Strains, Growth Conditions and Inoculum Preparation

*Salmonella* spp. strains (total 5) isolated from dairy products (*Salmonella typhimurium* and *Salmonella enterica* subsp. *enterica*) and one reference strain (*Salmonella enterica* subsp. *enterica* (D) ATTC 13076, MicroBioLogics, St. Cloud, MN, USA) were used to prepare a cocktail of *Salmonella* spp. strains which was used for the artificial contamination of fresh cheese samples. *Salmonella* spp. strains were isolated from food products and species confirmation was performed at the Department of Industrial and Food Microbiology (Faculty of Food Sciences, University of Warmia and Mazury in Olsztyn, Poland).

*Salmonella* spp. strains were stored individually at freezing temperature (−80 °C) in BHI broth (Merck, Warsaw, Poland) with 20% (*v*/*v*) glycerol (Merck, Warsaw, Poland), further grown by transferring 0.1 mL of the culture to 10 mL of BHI broth and incubated for 24 h at 37 °C (ICP500, Memmert GmbH + Co. KG, Schwabach, Germany). The second passage was performed by transferring 0.1 mL from each 24 h culture to 10 mL BHI broth followed again by incubation at 37 °C for 24 h. The initial concentration of microorganisms in each working subculture was determined by traditional plate counts method (BHI agar, Merck, Warsaw, Poland). The cocktail of *Salmonella* spp. strains was prepared by combining equal volumes (3 mL) of each activated strain. Estimated final concentration of *Salmonella* spp. in the cocktail was ca. 9 log cfu/mL.

### 2.3. Cheese Contamination and Storage

The cocktail of *Salmonella* spp. strains was decimally diluted to give a final concentration of ~6.5 log cfu/g of fresh cheese. Cheese samples (25 g) placed in sterile bags were inoculated with 100 µL of prepared cocktail of *Salmonella* spp. and stored at temperatures 5, 15 and 25 °C (ICP500, Memmert GmbH + Co. KG, Schwabach, Germany) for 33, 18 and 10 days, respectively. Sampling time depended on the applied storage temperature: 5 °C—every three days, 15 °C—every two days and 25 °C—each day.

### 2.4. Microbial Analysis

The number of *Salmonella* spp. was checked through analyses of three independent artificially contaminated cheese samples by using a selective medium—XLD agar (Merck, Warsaw, Poland). At each sampling the blender bags containing contaminated cheese were aseptically opened, followed by addition of 225 mL of a peptone water (Merck, Warsaw, Poland). Samples were homogenized in the lab blender BagMixer^®^ 400 W (Interscience, St. Nom la Bretèche, France). From each sample the decimal dilutions were made using 9 mL dilution blanks of peptone water. The enumeration of *Salmonella* spp. was performed in triplicate using 0.1 mL of the appropriate dilution, evenly distributed over the surface of the selective medium. After that, the plates were aerobically incubated at 37 °C for 48 h and enumerated. An average from 3 platings of each sampling point (cfu/g) was used to establish the growth kinetics of *Salmonella* spp. in the soft unripened cheese made from raw milk. The experiment was performed in 3 replicates for each studied temperature, using independent batches of soft unripened cheese.

Simultaneously to *Salmonella* spp. Enumeration, the number of coliforms and *E. coli* (Chromocult^®^ Coliform Agar), total aerobic microbial count (TSA with polysorbate 80 and lecithin), *S. thermophilus* (M 17 agar acc. to TERZAGHI), *Lactococcus* (MRS agar acc. ISO 15214) and *Enterobacteriaceae* (VRBD agar) was determined. The enumeration of yeasts and molds was performed using the TEMPO^®^ YM selective tests compatible with the TEMPO^®^ system (bioMérieux).

### 2.5. Physicochemical Measurements of the Soft Unripened Raw Milk Cheese

The chemical composition (protein, fat, dry matter, salt and water) of the unripened soft raw milk cheese was determined using a FoodScan (Foss, Warsaw, Poland). Water activity (aw) was measured using Hygropalm HP23-AW (Rotronic AG, Bassersdorf, Germany). The pH of the cheese was determined by using a Lab 860 electrode (Schott^®^ Instruments Inc., Mainz, Germany). The chemical composition and aw of cheese were measured at the beginning and at the end of the experiment, whereas the cheese pH was measured during the whole storage period at each temperature: at 5 °C—every three days, at 15 °C—every two days and at 25 °C—each day).

### 2.6. Kinetic Parameters of Salmonella spp. Survival in the Soft Unripened Raw Milk Cheese

The bacterial-count points transformed to log10 values were plotted against time (in days) in MS Excel v. 2010 (Microsoft). The following mathematical functions were used as primary models in order to determine the survival kinetics of *Salmonella* spp. in unripened soft cheese made from raw milk:

(a) Baranyi and Roberts model without lag [24,25] in Equations (1) and (2):

(1) $$N\left(t\right)=N_{0}+\mu_{\max}F\left(t\right)-\ln\left(1+\frac{e^{\mu_{\max}F\left(t\right)}-1}{e^{\left(N_{\max}-N_{0}\right)}}\right)$$

(2) $$F\left(t\right)=t+\left(1/v\right)\cdot \ln\left(e^{-\mathrm{vt}}+e^{-h_{0}}-e^{(-\mathrm{vt}-h_{0)}}\right)$$

where N(t)—cell concentration at time t (ln cfu/g), N_0_—initial cell concentration (ln cfu/g), N_max_—maximum cell concentration (ln cfu/g), μ_max_—maximum specific growth rate (1/h), v—rate of increase in limiting substrate and h_0_—product of μmax·λ, where λ denotes the duration of the lag phase in days.

The Baranyi and Roberts model without lag was fitted to the observed survival data of *Salmonella* spp. in fresh soft raw milk cheese by using the DMFit MS Excel add-in (Food Safety Centre, Hobart, Australia), which is implemented in the ComBase database (www.combase.cc (accessed on 27 April 2021))—an important and valuable tool in predictive microbiology application. The database includes more than 60,000 records on the behavior of microorganisms in different environments (ComBase Browser), as well as predictive models to predict growth and inactivation (ComBase Predictor) [23].

(b) Weibull model [26], in Equation (3):

(3) $$\log_{10}N\left(t\right)=\log_{10}N_{0}-{\left(t/\delta \right)}^{p}$$

where N_0_—initial population, parameter p—characterizes the shape of the curves and δ—time for the first decimal reduction in microbial population (10-fold reduction in the surviving population).

The freeware add-in GInaFiT v. 1.7 [27] was used as an add-in Excel component for the fitting procedure of the Weibull and log-linear models and the determination of the statistical indices.

The statistical analyses embraced the determination of the mean square error (MSE) and the correlation coefficient (R^2^). Both of these parameters were automatically reported by the GInaFiT and DMFit tools.

An analysis of variance (F-test two samples for variances) was used for analyses of the data (MS Excel). The obtained results were considered significant at *p* < 0.05.

## 3. Results

### 3.1. Physicochemical Characteristics of Fresh Raw Milk Cheese during Storage

**Figure 1:**
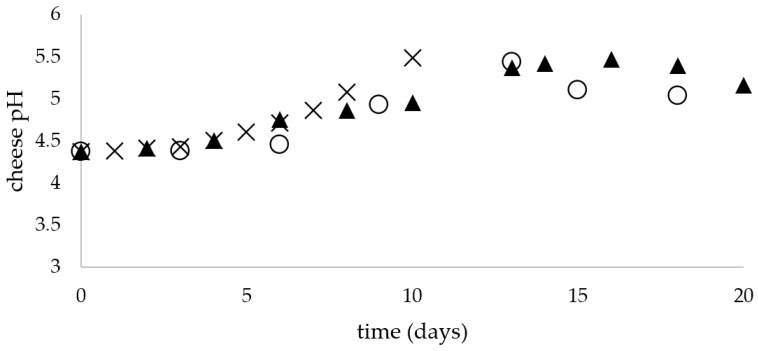
Changes in unripened soft raw milk cheese during storage at 5 (○), 15 (▲) and 25 (x) °C.

The analyzed composition of the unripened soft raw milk cheese was as follows: proteins—15.05 ± 0.32%, dry matter—28.75 ± 0.26%, fat—9.35 ± 0.14% and water—71.25 ± 1.14%. The dry matter of tvarog consisted mainly of protein and fat (final fat content depended on fat standardization of cheese milk, if any) and lactose. Carbohydrate content (mainly lactose) was approx. 4% in tvarog. Ash content was in the range of 0.7 to 1.0%. The measured aw values of the unripened soft raw milk cheeses remained constant during storage (0.975 ± 0.005) without statistically significant differences (*p* > 0.05), irrespective of applied storage temperature. Figure 1 shows the changes of the pH value of the soft unripened raw milk cheese stored at 5, 15 and 25 °C. The measured increase in the pH during storage from the initial value 4.37 was 0.665 ± 0.04 at 5 °C, 0.795 ± 0.06 at 15 °C and 1.12 ± 0.08 at 25 °C.

### 3.2. Microbial Characteristics of Fresh Raw Milk Cheese during Storage

**Figure 2:**
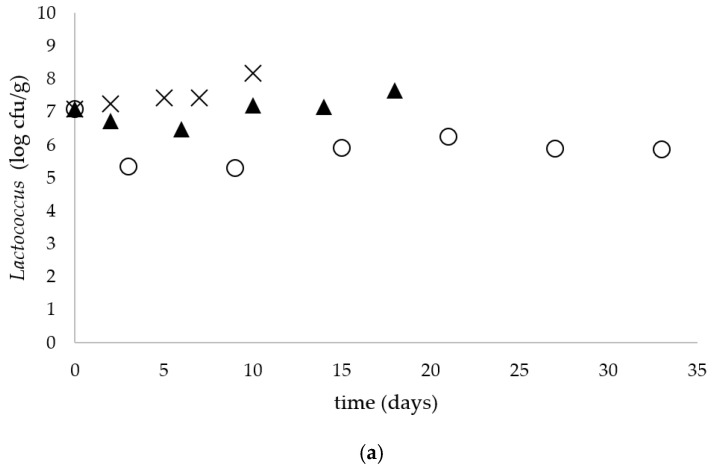

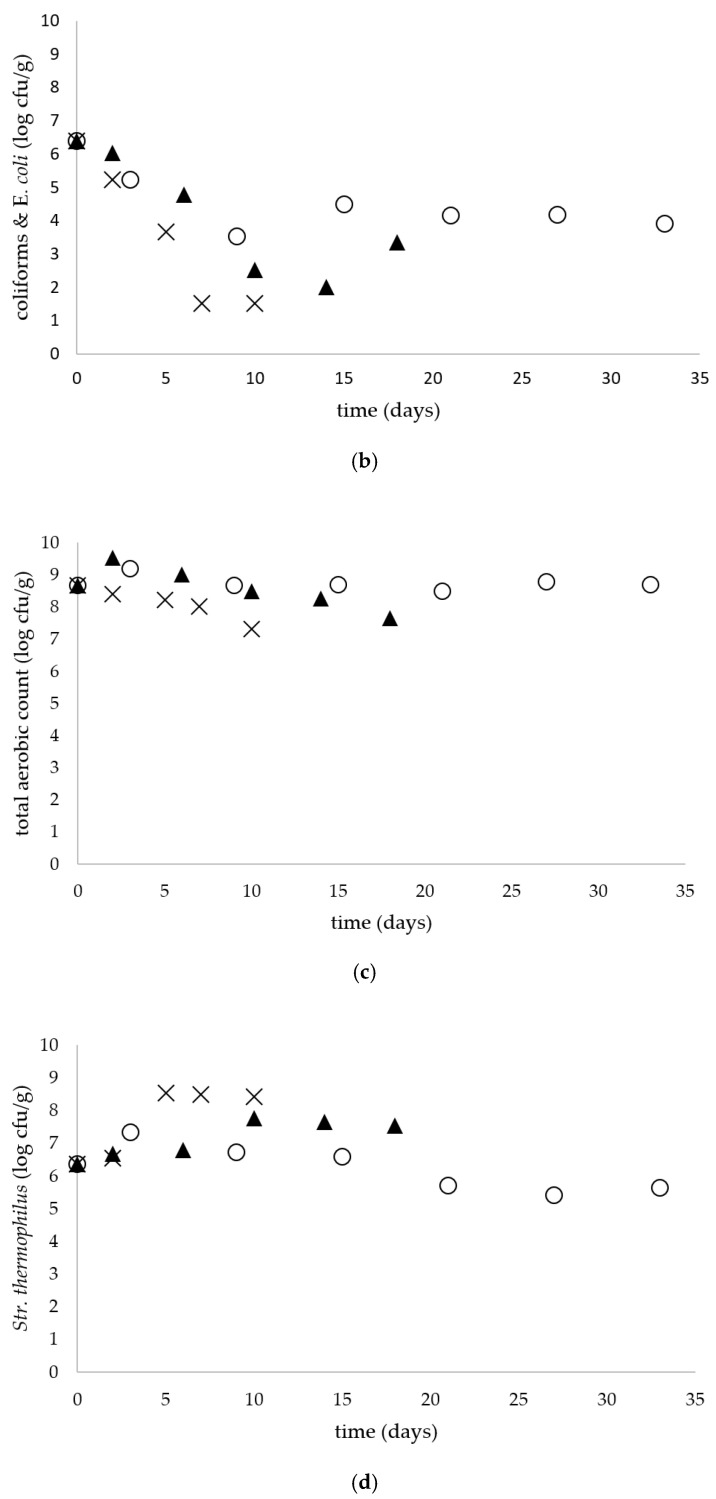

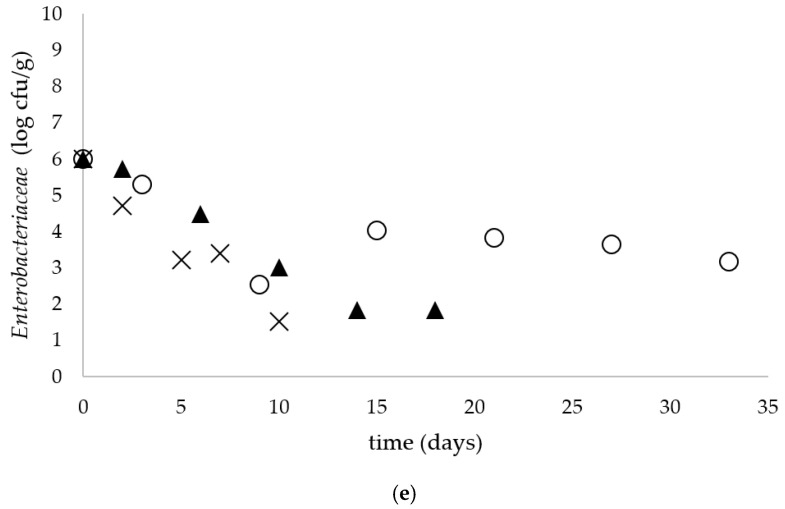
Fate of: (**a**) *Lactococcus*, (**b**) coliforms and *E. coli*, (**c**) total aerobic count, (**d**) *S. thermophilus* and (**e**) *Enterobacteriaceae* in soft unripened cheese made from raw milk at 5 (○), 15 (▲) and 25 (x) °C.

The yeasts and molds population remained constant (5.7 ± 0.2 log cfu/g) in the soft unripened raw milk cheese during storage at all tested temperatures. Figure 2a–e shows the changes in the microbial population of the experimentally contaminated raw milk cheese during storage at 5, 15 and 25 °C. A decrease in the number of *Lactococcus* (Figure 2a) from the initial level 7.1 log cfu/g by 1.21 log was observed at 5 °C. However, an increase of 0.57 and 1.1 log cfu/g of *Lactococcus* in fresh unripened raw milk cheese was noticed during storage at 15 and 25 °C, respectively (Figure 2a). A decrease in the *S. thermophilus* population by 0.7 log cfu/g in fresh unripened raw milk cheese at 5 °C was observed (Figure 2d). At higher storage temperatures, however, the level of *S. thermophilus* increased by 1.2 and 2.05 log cfu/g during storage at 15 and 25 °C, respectively. The number of Enterobacteriaceae decreased by 2.8, 4.2 and 4.5 log cfu/g in the fresh unripened soft cheese during storage at 5, 15 and 25 °C, respectively (Figure 2e).The number of coliforms and *E. coli* reduced by 2.47, 3.05 and 4.87 log cfu/g in fresh unripened raw milk cheese during storage at 5, 15 and 25 °C, respectively (Figure 2b). Figure 2c shows the changes in the total aerobic count during storage in fresh unripened cheese made from raw milk. The total aerobic count during storage for 33 days at 5 °C remained at the level of 8.6 log cfu/g, while at higher storage temperatures a decrease in the number of bacteria was noticed by 1.0 and 1.35 log cfu/g at 15 and 25 °C, respectively.

### 3.3. Survival of Salmonella spp. in Fresh Raw Milk Cheese during Storage

**Figure 3:**
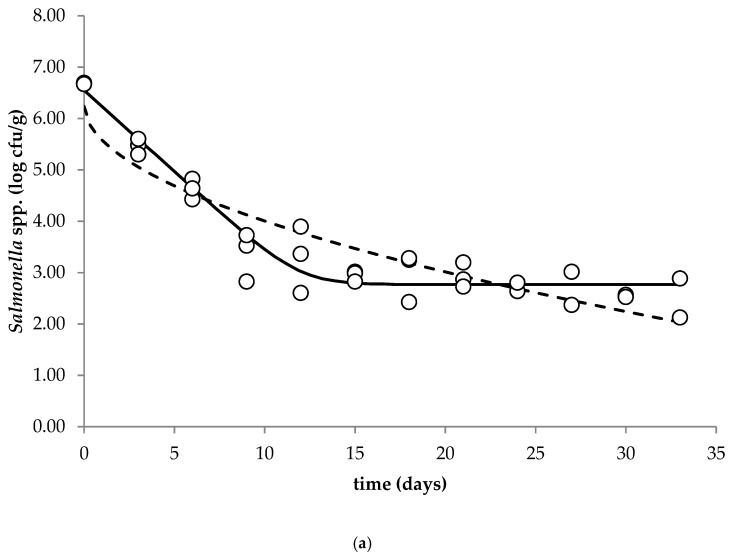

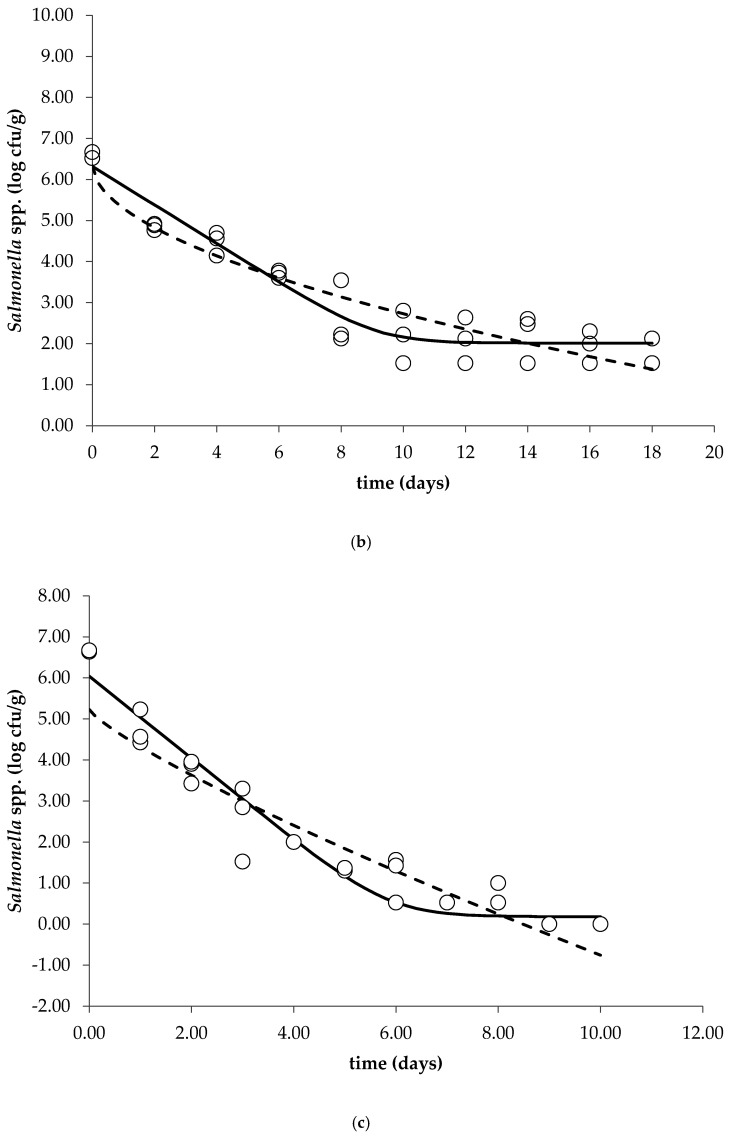
Fitted Baranyi and Roberts model (no lag) (solid line) and Weibull model (dashed line) to the observed behavior of *Salmonella* spp. (○) in soft unripened cheese made from raw milk at 5 (**a**), 15 (**b**) and 25 (**c**) °C.

The survival of *Salmonella* spp. in the soft unripened raw milk cheese during storage at 5, 15 and 25 °C is shown in Figure 3.

A decrease in the concentration of pathogen was observed, and reached the final number dependent on the applied storage temperature. The population of *Salmonella* spp. in the cheese decreased from the initial concentration 6.67 log cfu/g by 4.16 and 4.85 log cfu/g at 5 and 15 °C, respectively. At the highest applied storage temperature, namely 25 °C, the number of pathogens in raw milk cheese was below the detection limit of the applied analytical method after 7 days of the experiment. By observing the graphs of the survival curves it can be observed that the population of *Salmonella* spp. (sensitive population) was rapidly inactivated (the higher the storage temperature, the higher the inactivation rate), followed by a slowing down in inactivation (tailing effect; resistant population).

In the present study the kinetic parameters that characterized the behavior of the *Salmonella* spp. were estimated by Baranyi and Roberts (without lag) and Weibull models (Table 1).

Statistically significant differences (*p* < 0.05) were observed for the values of maximum specific death rate µ_max_ (1/h) estimated by the Baranyi and Roberts model (without lag) and for the δ parameter estimated by the Weibull between all studied temperatures (5, 15 and 25 °C). The goodness-of-fit of the primary models (Table 2) was tested by calculating the statistical indices, namely R^2^ and MSE.

On this basis it was concluded that the Baranyi and Roberts model (without lag) gave the more adequate description of the survival of *Salmonella* spp. in the fresh soft raw milk cheese during storage at 5, 15 and 25 °C. Figure 3 presents fitted Weibull and Baranyi and Roberts (no lag) models for the observed growth of *Salmonella* spp. in the contaminated unripened soft raw milk cheese at 5, 15 and 25 °C. Although the growth of *Salmonella* spp. was inhibited during storage at all tested temperatures, the microorganisms survived for an extensive period. The number of microorganisms at the end of the experiments was still high, especially at 5 °C, and when the initial contamination of the unripened soft raw milk cheese is high, it can pose a threat to public health.

## 4. Discussion

The conditions for the hygienic performance of the production process are the cleanliness of the equipment constituting the production line, the use of microbiologically clean technological water and hygienic conditions for packaging and storing the product at the distributor. The chemical composition of tvarogs, i.e., fat, protein, lactose and high water content, make them an excellent medium for bacteria growth. Thus, microorganisms that have been deliberately introduced into milk or come from secondary contamination may also be responsible for the limited shelf-life of tvarog cheeses. In order to meet the highest hygiene standards, it is important to ensure an effective cleaning procedure, together with hygienic conditions for packaging and storage of the product [28].

Chojnowski et al. (2010) [28] studied changes in the chemical composition of unripened soft cheese during storage under refrigeration conditions and did not observe any changes in the composition. Similar findings were obtained in the present study; the composition of the fresh raw milk cheeses was stable during storage in terms of protein, fat, dry matter, salt and water content during the entire storage period at 5, 15 and 25 °C.

Changes in pH during storage of tvarog cheese rely mainly on the activity of LAB. Dmytrów et al. (2007) studied the influence of two different packaging materials on selected quality features of tvarog cheese during storage and showed that there was a slight decrease in pH value (approx. 4.4) during the first 7 days of cold storage (5 °C), and then a slight increase (4.5), with the final value (after 21 days) not exceeding the beginning value (4.7) [29]. Godula et al. (2018) showed slight increase in pH value of traditional and lactose-free tvarogs, from 4.17 to 4.33 and 3.98 to 4.11, respectively [30].

The increase in pH value for experimental cheeses in this study may have resulted from a gradual inhibition of the activity of lactic streptococci or even their partial extinction during storage. Studies of Cais and Wojciechowski (1996) demonstrated a sharp drop in acidity after the third day of storage of the tvarog, following a previous increase in acidity (drop in pH), which the authors related to casein hydrolysis. The decrease in acidity was accompanied by an increase in pH and fermentation of lactose [31]. Activity of bacterial enzymes changes over time, which may result in protein hydrolysis. *Lactococcus lactis* bacteria have the ability to break down casein due to serine proteinase anchored in the cytoplasmic membrane. Extracellularly secreted enzymes anchored in the cell membranes of the starter cultures are considered to be the main source of peptidases in cheese that are involved in the hydrolysis of oligopeptides [32].

Milk is an excellent reservoir for the growth of microorganisms, as it contains basic nutrients (proteins, fat, lactose, vitamins and minerals) necessary for their growth. Milk is constantly exposed to the action of microorganisms, i.e., during milking, storage, transport and processing. Lactic acid bacteria and other microorganisms present in raw milk play an important role in the development of the desired flavor as well as the physical properties of cheeses made of ram milk. However, it is possible that pathogenic bacteria and toxins produced by them are present in raw milk, which may pose a health hazard. Moreover, spoilage microorganisms (or their metabolites) can also contribute to cheese defects and spoilage [14].

Berthlod et al. (2007) detected yeast in 65% of cheese (tvarog) samples tested at the level from 1.4 to 4.6 log cfu/g, while in the remaining 35% of samples analyzed, yeast were not present in 0.1 g of the product. The presence of molds was detected in 45% of tvarog samples ranging from 0.7 to 2.3 log cfu/g, and the remaining 55% of samples did show the presence of molds in 0.1 g of the product [33]. In another study, it was observed that the number of yeast in the vacuum- and non-vacuum-packed tvarogs increased during refrigerated storage, and after 14 days it reached the level of 6 log cfu/g in 1 g of tvarog [34]. In our study the detected number of yeasts and molds (5.7 log cfu/g) was higher when compared to the aforementioned studies. Due to the lack of a pasteurization process, all bacteria present in raw milk are transferred to the final product. On the contrary to ripened raw milk cheeses, unripened cheeses (e.g., tvarogs) in general have a limited shelf-life, as the spoilage of these products may occur faster compared to products made from pasteurized milk.

The presence of coliform bacteria in raw milk is caused by improper hygienic conditions during the milking and farm environment. Additionally, the presence of coliform bacteria can serve as an indicator of the hygienic condition of the dairy plants. In a study conducted by Berthlod et al. (2007) where the microbiological quality of tvarogs from 15 manufacturers was determined, the presence of coliform bacteria was found in 45% of the tested samples [3]. In our study the presence of coliform bacteria was observed in all raw milk cheese samples with the initial level 6 log cfu/g. The low pH of the product and the presence of LAB were probably the main factors that caused a decrease in coliforms and *Enterobacteriacae* in tvarog during storage. The high loads of coliform bacteria detected in raw milk cheese could be due to improper hygiene of the production practices. The manufactures should applied in terms of good manufacturing practices in order to prevent the increase in microbial loads.

Traditional tvarog cheeses are naturally characterized by a high total microbial count. In a study of Berthlod et al. (2007) where a microbiological analysis of 20 samples of tvarog cheeses was carried out, the total bacterial count (TBC) ranged from 4.6 to 8.34 log cfu/g. Moreover, for 26% of the tested samples of tvarog, TBC was at the level of <6 log cfu/g, for 5% of the samples it was within the range of >6 to 7 log cfu/g and for 52% of the samples TBC was in the range >7 to 8 log cfu/g. The TBC of three cheese samples slightly exceeded 8 log cfu/g [3]. In our study the total microbial count at 5 and 15 °C remained at the level above 8 log cfu/g during the entire storage period, whereas at 25 °C the count decreased from the initial level of 8.6 to 7.3 at the end of storage period.

Apart from the native microflora of the raw milk, during tvarog production the lactic acid bacteria (*Lactococcus lactis* subsp. *lactis*, *Lactococcus lactis* subsp. *cremoris* and *Lactococcus lactis* subsp. *lactis biovar diacetylactis*) are added as starter cultures, which exhibit an antagonistic mechanism against *Salmonella*. The presence of lactic acid bacteria (LAB) causes pH decline in cheese curd during fermentation, which negatively affects the growth potential of foodborne pathogens as a result of competition for nutrients and production of antimicrobial compounds, including bacteriocins. The identified factors which influence the extent of competition and production of bacteriocins by LAB are, among others, temperature and pH [7]. In the present study, the *Lactococcus* concentration throughout the whole experiment was in a range from 5.8 to 8.2 log cfu/g. Yoon et al. (2016) observed a growth inhibition of foodborne pathogens by native lactobacilli, lactococci and enterococci by production of bacteriocins and other antimicrobial substances, e.g., hydrogen peroxide and organic acids [7]. The microbial competition and the presence of LAB added as starter culture, in combination with the low pH of the fresh unripened cheese made from raw milk, can explain the growth inhibition and high inactivation rates of *Salmonella* spp. during storage at 5, 15 and 25 °C observed in the present study.

The findings obtained in the present study correspond to other studies where the behavior (growth, survival and inactivation) of *Salmonella* spp. in the raw milk cheeses was studied. Tamagnini et al. (2005) studied the behavior of *Yersinia enterocolitica* and *Salmonella typhimurium* in Crottin goat’s cheese stored at 5, 15 and 25 °C. It was found that the growth of pathogens was inhibited during storage, although bacteria survived for an extensive period and the counts at the end of the experiment at 5 and 15 °C were high, indicating that contamination with high bacterial numbers represents a potential health hazard [34].

The main objective of primary modeling is to determine how the microbial population changes over time by selecting the best-fit parameters of the bacterial behavior (growth, survival and inactivation). A decision regarding the most appropriate model is a crucial process, as fitting an inappropriate model to the data can result in wrong predictions of the microbial behavior. The obtained survival curves of *Salmonella* spp. in the fresh soft raw milk cheese during storage at 5, 15 and 25 °C were subjected to primary modeling. According to Mataragas et al. (2008), models capable of describing the biphasic survival of the pathogen are the Geeraerd, Cerf, Albert¬–Mafart, Whiting, Zwietering and Baranyi models [35]. Oliveira et al. (2013) applied successfully the Weibull model to fit survival curves and determine the inactivation kinetics of *Salmonella enterica* subsp. *enterica* in thermal and non-thermal processes [36]. Performed within the present study, microbiological analysis confirmed that the storage temperature significantly influenced the inactivation rate of *Salmonella* spp. in soft unripened cheese produced from raw milk during storage at 5, 15 and 25 °C. In cheese samples stored at 25 °C, inactivation of *Salmonella* proceeded faster compared to the remaining storage temperatures. It was concluded that due to the possible exposure of the consumer to the *Salmonella* spp. through cheeses made from raw milk, appropriate risk communication on the consumption of these products, in particular to a vulnerable population, is recommended. Obtained in the present study, the results suggest that contamination of raw milk cheese by *Salmonella* might pose a risk for consumer health, especially for a vulnerable population. The survival kinetics of *Salmonella* spp. in soft unripened cheese during storage calculated in the present study can be incorporated into quantitative risk assessment studies in order to estimate the threat to consumers regarding the presence of *Salmonella* in soft unripened raw milk cheese.

## Figures and Tables

**Table 1:** Kinetic parameters (means ± SD) of the Baranyi and Roberts model (no lag) and Weibull model for the fate of *Salmonella* spp. in soft unripened cheese made from raw milk at 5, 15 and 25 °C.

T (°C)	Baranyi and Roberts			Weibull		
	N_0_	N_max_	µ_max_	δ	*p*	N_0_
5	6.54 ± 0.17	2.76 ± 0.08	−0.316 ± 0.03	2.20 ± 1.73	0.53 ± 0.14	6.24 ± 0.19
15	6.32 ± 0.24	2.01 ± 0.14	−0.471 ± 0.05	0.93 ± 0.66	0.54 ± 0.12	6.34 ± 0.41
25	6.04 ± 0.31	0.18 ± 0.22	−0.998 ± 0.11	1.13 ± 0.73	0.82 ± 0.22	5.23 ± 0.62

where N_0_—initial cell concentration (log cfu/g), N_max_—final cell concentration (log cfu/g), µ_max_—maximum specific death rate (1/h), δ—Weibull parameter and *p*—Weibull parameter.

**Table 2:** Statistical indices for the Baranyi and Roberts model (no lag) and Weibull model used to fit *Salmonella* spp. in soft unripened cheese made from raw milk at 5, 15 and 25 °C.

T (°C)	Parameter	Baranyi	Weibull
5	R^2^	0.97	0.91
	MSE_model_	0.20	0.20
15	R^2^	0.97	0.20
	MSE_model_	0.07	0.17
25	R^2^	0.96	0.92
	MSE_model_	0.41	0.45

## Data Availability

The data presented in this study are available on request from the corresponding author.
